# Medium-term clinical results in the treatment of supracondylar humeral fractures in children: does the surgical approach impact outcomes?

**DOI:** 10.1186/s10195-024-00781-3

**Published:** 2024-09-11

**Authors:** Elena Manuela Samaila, Ludovica Auregli, Lorenzo Pezzè, Gabriele Colò, Bruno Magnan

**Affiliations:** 1 Department of Orthopaedics and Trauma Surgery, University of Verona, Verona, Italy; 2 Department of Orthopaedics and Trauma Surgery, Azienda Ospedaliera Universitaria Ss. Antonio e Biagio e Cesare Arrigo, Alessandria, Italy

**Keywords:** Supracondylar, Humerus, Fracture, Treatment, Pediatric, Outcome

## Abstract

**Background:**

Recent literature has found a consensus in favor of conservative treatment for type II supracondylar humeral fractures (SCHF). This retrospective observational study compares the short- to medium-term functional outcomes of conservative versus surgical treatment in 31 patients with SCHF (Gartland II and III) to assess the potential superiority of one approach over the other.

**Materials and methods:**

Thirty-one pediatric patients treated for SCHF—19 classified as Gartland II and 12 as Gartland III—were assessed in our department. Eight patients underwent closed reduction and cast immobilization, 22 were treated with closed reduction and percutaneous pinning, and one underwent open reduction and internal fixation with plates. Clinical and functional data were collected during follow-up, including elbow and forearm range of motion (ROM), grip strength, carrying angle, Flynn’s criteria, and Disabilities of the Arm, Shoulder, and Hand (DASH) score.

**Results:**

The average follow-up was 3.3 years (± 1.4 years). All patients demonstrated good functional recovery. According to Flynn’s criteria, 85% and 81% of the patients achieved a satisfactory outcome in elbow flexion and carrying angle, respectively. No cases of nerve injuries were reported.

Four patients developed cubitus varus in the Gartland II group, which was treated with closed reduction and casting with the initial alignment maintained (without a loss of reduction during the first week). However, compared to this group that was conservatively treated, functional and clinical outcomes were significantly better in the group with SCHF Gartland II treated with reduction and pinning (*p* < 0.05).

**Conclusions:**

Although some recent studies have demonstrated positive outcomes with conservative treatment for both Gartland IIA and IIB fractures, the short- to medium-term functional results in our study emphasize that superior outcomes were obtained with surgical treatment for Gartland II fractures when compared to those treated conservatively.

*Trial registration*: This study was performed in line with the principles of the Declaration of Helsinki. Ethics approval was obtained from our institute’s ethics committee (registry no. 3511).

*Level of evidence*: Therapeutic level III

## Introduction

Supracondylar humeral fractures (SCHF) are the most common elbow injuries in children, accounting for approximately 60% of all elbow fractures in this age group [1]. Epidemiological data in the United States indicate an annual incidence ranging from 60.3 to 71.8 per 100,000 children, with a peak occurrence between the ages of 3 and 6 years [2]. The Wilkins-modified Gartland classification [3, 4] is widely used to categorize supracondylar humeral fractures. Type I (G I) identifies a nondisplaced fracture, while type II (G II) refers to fractures displaced anteriorly with a posterior cortical contact. Type II further includes two subgroups: type II A, which is stable with no rotation, and type II B, which involves translation or rotation of the distal fragment. Gartland type III (G III) includes displaced fractures without cortical contact.

SCHF can be managed with several treatment strategies, depending on the degree of displacement and potential complications. However, currently, there are no universally recognized guidelines [5]. In 2012, the American Academy of Orthopaedic Surgeons (AAOS) guidelines suggested closed reduction with pin fixation as the recommended approach for managing displaced pediatric Gartland II and III supracondylar fractures [6]. Furthermore, in 2014, AAOS developed Appropriate Use Criteria (AUC) for managing pediatric SCHF based on 220 patient scenarios. Their case studies illustrate how the AUC can be integrated into the decision-making process, and they recommended pinning for almost all type II SCHF [7, 8]. However, recent works have criticized the surgical approach for all type II SCHF: Silvia et al. challenge the AUC recommendations and propose nonoperative management for Gartland IIA SCHF (without rotational or coronal malalignment) [9]; other authors found no distinctions in clinical or radiological outcomes between conservatively treated type IIA and type IIB fractures and, similarly, no differences, both clinically and radiologically, when comparing conservative management with surgical management for type IIB fractures treated initially with closed reduction and casting [10].

The complications of SCHF in pediatric patients can be divided into acute and chronic. Acute complications include the risk of compartment syndrome, vascular complications due to brachial artery injury (such as pulseless pink hand or ischemic hand), and neurological deficits. Chronic complications include malunion leading to cubitus varus or cubitus valgus [11, 12].

In response to the absence of a definitive consensus regarding the management of Gartland II fractures, this retrospective study aims to assess and compare the short- to medium-term functional outcomes of SCHF based on the degree of displacement (G IIa, G IIb, and G III) and treatment type (closed reduction and casting or reduction–fixation with K-wires) and to elucidate any potential superiority of one approach over the other.

## Materials and methods

Thirty-one pediatric patients treated for SCHF between 2012 and 2018 were evaluated. Written informed consent was obtained from the participants' parents. Inclusion criteria comprised monotrauma supracondylar fractures (G II and III; International Classification of Diseases 812.41) in children under 16 years old at the time of fracture. Exclusion criteria included nondisplaced fractures (G I), follow-up periods shorter than 1 year, patient refusal to participate in the study, and polytrauma. This study was performed in line with the principles of the Declaration of Helsinki. Ethics approval was obtained from our institute’s ethics committee (registry no. 3511).

**Fig. 1:**
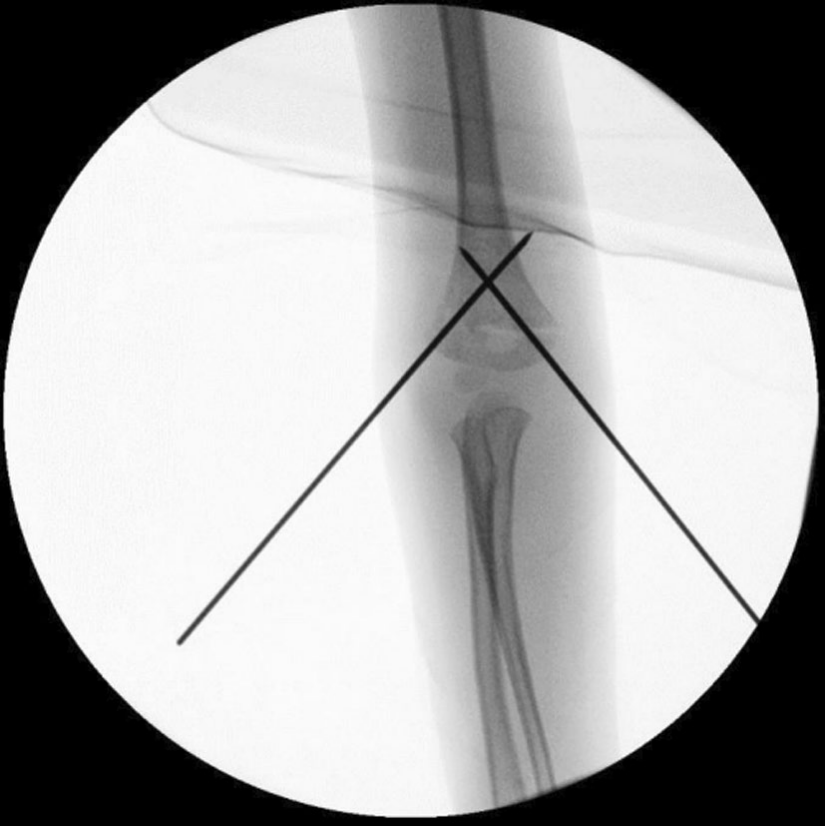
Intraoperative antero-posterior radiograph: reduction and pinning fixation with two crossed pins

**Fig. 2:**
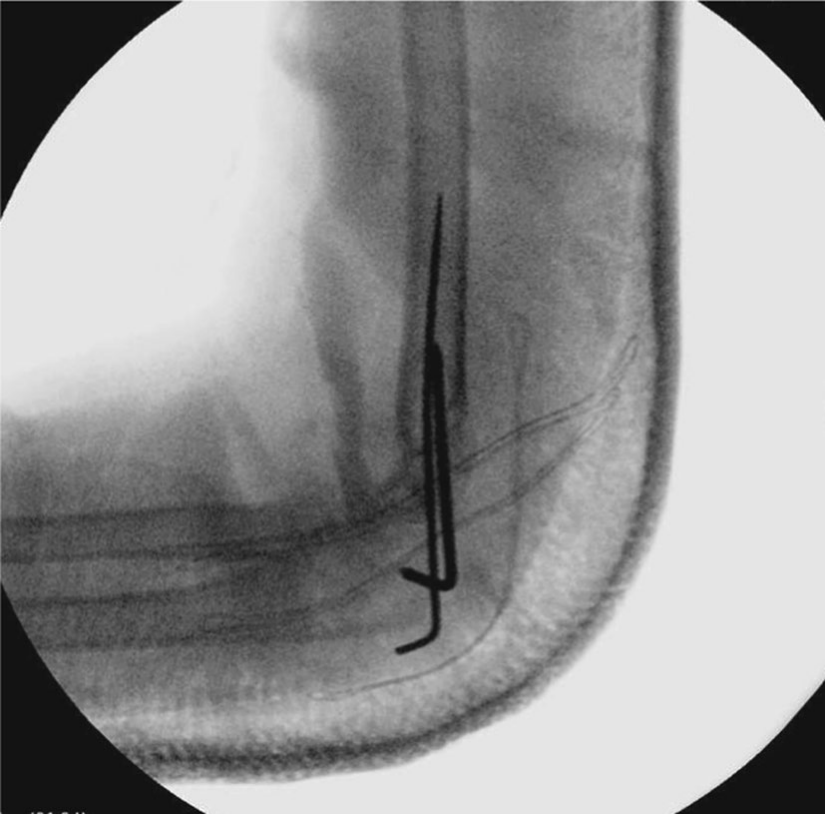
Intraoperative lateral view radiograph: reduction and pinning fixation with two crossed pins

The study included 21 males and 10 females, with an average age at the time of fracture of 6.8 ± 2.3 years (6.0 ± 2.1 for males and 8.6 ± 1.7 for females). The fracture occurred in the left arm, the nondominant side, in 68% of patients, consistent with epidemiological findings in the literature [13]. We categorized patients into two groups based on the Wilkins-modified Gartland classification: 19 fractures were classified as type II, with nine classified as type IIa and 10 as type IIb, while 12 fractures were classified as type III. We managed the fractures according to the severity of displacement: nine patients with G IIa fractures underwent closed reduction with sedation followed by casting, but one patient from this group exhibited a loss of reduction during the follow-up 1 week after casting and needed surgical reduction and fixation with K-wires. In the remaining 22 patients, 10 had G IIb fractures, while 12 had G III fractures; all of them necessitated surgical intervention. Among this group, 18 patients underwent closed reduction followed by fixation with two crossed pins (see Figs. 1 and 2), one patient underwent fixation with three crossed pins (two lateral and one medial), two patients were treated with two lateral and parallel K-wires, and one patient underwent fixation with a plate and screws (Table 1).

**Table 1:** Types of fracture and treatments

Type of fracture	Total no. of patients	Closed reduction and casting	Two crossed pins	Three crossed pins	Two parallel pins	Orif with plates
G IIa	9	8	1			
G IIb	10		8		2	
G III	12		10	1		1

*Orif* open reduction and internal fixation

A total of five orthopedic surgeons participated in the surgical procedures.

**Fig. 3:**
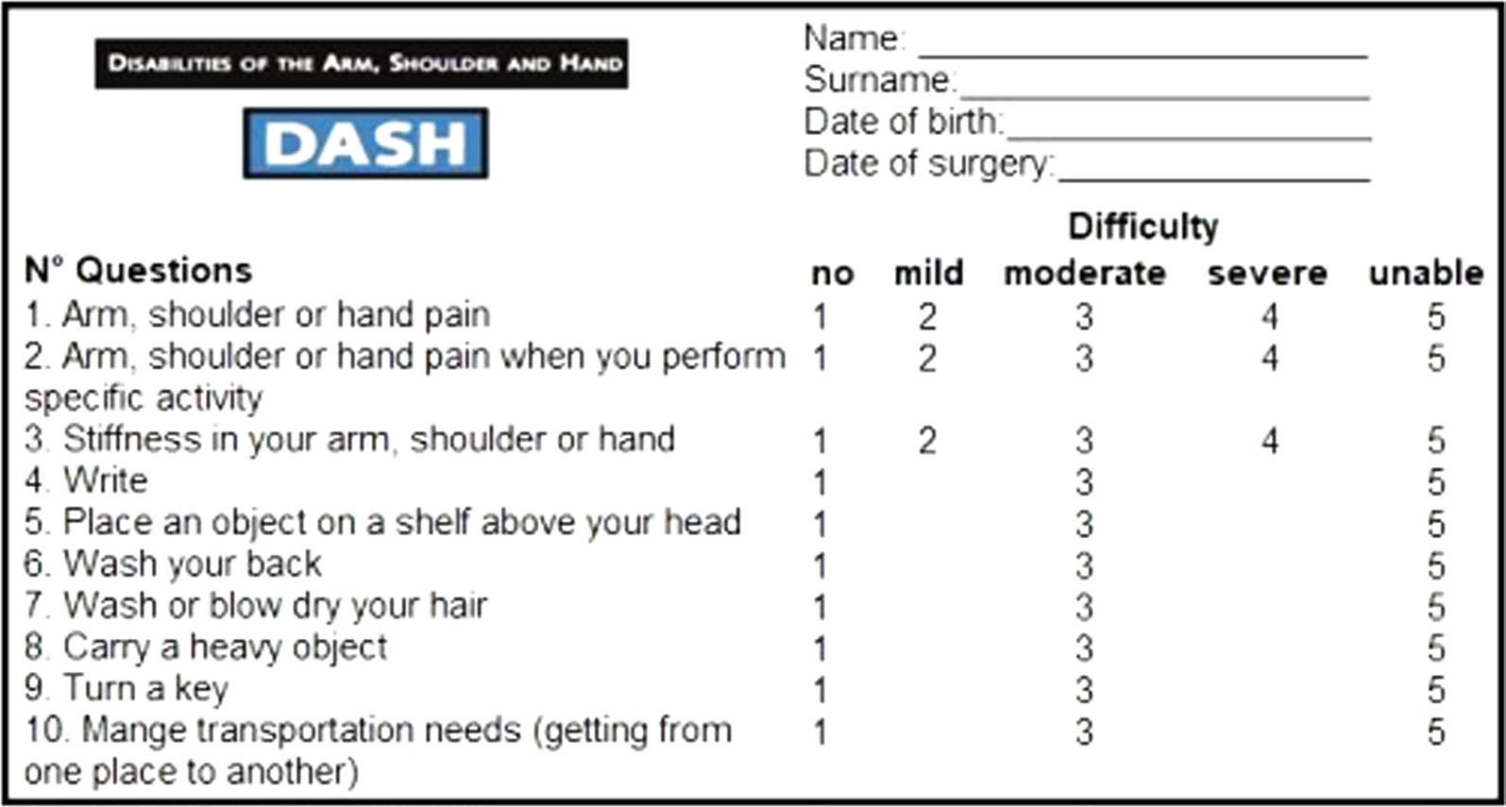
m-DASH questionnaire

Our study utilized a modified version of the the DASH (Disabilities of the Arm, Shoulder, and Hand; m-DASH) questionnaire, tailored for pediatric use. This version comprises 10 questions. By assessing pain, stiffness, and difficulties in performing daily activities, the questionnaire aims to provide an estimated score reflecting the level of disability caused by the injury. The questionnaire consists of five response options for the first three questions (none, mild, moderate, severe, or unable), and three response options for the remaining questions (none, moderate, or severe). The number of response options is reduced due to children's difficulty in differentiating between various degrees of movement difficulty (Fig. 3).

All 31 patients were evaluated using the m-DASH score, but only 27 patients consented to follow-up assessments, which included measurements of elbow range of motion (ROM), carrying angle, and grip strength. These metrics were assessed using a goniometer and a digital hand dynamometer (Kyto Fitness Technology, Guangdong, China; grip strength measurement of 200 lbs/90 kgs hand grip) and compared with those of the contralateral normal arm.

Normal values for flexion and extension in adults range from 140° to 150° and from 0° to 5°, respectively. In our pediatric sample, we calculated the mean, standard deviation, and confidence interval for the healthy limb to establish a specific range of normal values. Similarly, we applied this process to extension, pronation, supination, and carrying angle. Based on the statistical analysis conducted, the reference normal value (confidence interval) for elbow flexion is 140–144°; for extension, it ranges between − 6° and 0°; for pronation, 89–93°; and for supination, 92–99°. Finally, for the cubital angle, the specific normal values for our sample are 12–16° of valgus.

Flynn’s criteria [14] were employed to categorize the outcomes of SCHF. These criteria consider residual deformity and functional factors separately, based on the loss of carrying angle and flexion reduction. In their 1976 study, Flynn et al. evaluated cases of supracondylar fracture over a 16-year period and established criteria to determine whether the difference in angle between the fractured and healthy elbows can be considered satisfactory or unsatisfactory. According to this classification, the results are deemed excellent when the difference in angle between the fractured and healthy elbows falls between 0° and 5°, good when it is between 6° and 10°, and a fair/modest recovery when the angle difference ranges from 11° to 15°. A difference greater than 15° is considered poor (Table 2). Patient outcomes, according to Flynn's criteria, were correlated with treatment and fracture type.

**Table 2:** Flynn’s criteria

Result	Rating	Loss of carrying angle (°)	Flexion reduction (°)
Satisfactory	Excellent	0–5	0–5
	Good	> 5–10	> 5–10
	Fair/modest	> 5–10	> 5–10
Unsatisfactory	Poor	> 15	> 15

The medium-term results were analyzed using the healthy side of the same patient as a control, as it exhibited no morbidity significant enough to affect statistical measurements. To assess differences in continuous variables such as angles and strength, Student's* t*-test was employed. The analysis encompassed all fractures, with the healthy limb compared with the fractured one. Subsequently, clinically observed functional outcomes were correlated with different treatment modalities. The study's significance threshold was set at a* p* value < 0.05 (a* p* value < 0.01 was highly significant). The theory underlying the* t*-test aims to reject the null hypothesis and thus to suggest that the difference in data, such as the angle disparity, is not due to chance but is significantly influenced by the fracture's impact on clinical data. Data analysis was performed using the statistical software SPSS Statistics.

## Results

**Fig. 4:**
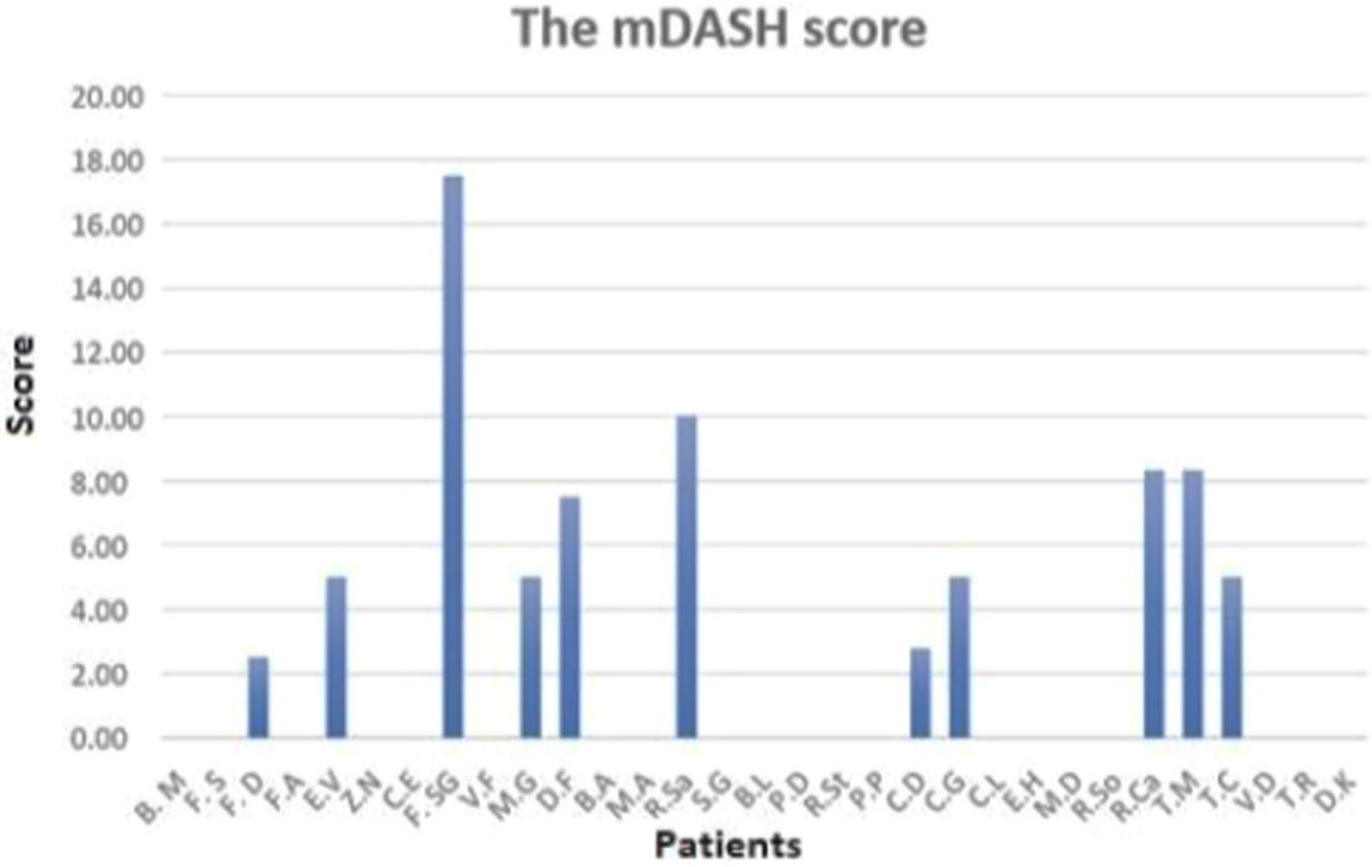
m-DASH results

The average duration of follow-up was 3.3 ± 1.4 years. The m-DASH score indicated favorable outcomes for most of our cohort; however, 11 patients experienced varying degrees of difficulty performing certain activities (Fig. 4).

The clinical results were initially analyzed collectively without distinguishing between treatment variations or fracture types. Evaluation based on Flynn’s criteria, range of motion (ROM)—flexion, and carrying angle yielded the following insights: regarding elbow ROM, according to Flynn’s criteria, four patients (15%) exhibited a poor medium-term outcome, while 66% achieved good (18%) or excellent (48%) results and 19% obtained average scores. The mean for the uninjured side is 142° ± 4.3°, while that for the fractured side is 137° ± 9.7°; this indicates a significant decrease in flexion angle (mean difference of 5° ± 8°), as confirmed by a Student’s* t*-test analysis (*p* value < 0.01) (Table 3).

**Table 3:** ROM and Flynn’s criteria

Patients		ROM		Rating (Flynn's criteria)
		Uninjured side	Fractured side (N.V. 140–144°)	
1	B.A	140°	140°	Excellent
2	B.M	140°	142°	Excellent
3	B.L	140°	146°	Good
4	C.L	145°	145°	Excellent
5	C.E	142°	140°	Excellent
6	C.G	136°	115°	Poor
7	D.F	140°	125°	Poor
8	E.H	145°	135°	Fair
9	E.V	142°	125°	Poor
10	F.S	150°	145°	Good
11	F.D	150°	150°	Excellent
12	F.SG	140°	135°	Good
13	F.A	139°	145°	Good
14	M.A	135°	125°	Fair
15	M.D	145°	135°	Fair
16	M.G	142°	140°	Excellent
17	P.P	140°	140°	Excellent
18	P.D	140°	140°	Excellent
19	R.So	143°	145°	Excellent
20	R.Sa	140°	115°	Poor
21	R.St	141°	140°	Excellent
22	S.G	138°	124°	Fair
23	T.M	150°	135°	Fair
24	T.C	144°	140°	Excellent
25	V.F	135°	142°	Good
26	Z.N	145°	145°	Excellent
27	T.R	150°	150°	Excellent
Average	142°	137°		
SD	*4.34*	*9.68*		
*p* value	***0.0054***			

Bolditalic value indicates statistically significant *p* value

*N.V.* Normal value

Regarding the carrying angle, 17 patients (63%) attained a good/excellent outcome, while five patients (18.5%) experienced fair outcomes and five showed poor results. The average difference between the unaffected side (with a carrying angle of 14° ± 5.6°) and the injured side (8° ± 8.9°) was 6° ± 8° (*p* < 0.001). Nine children displayed a discrepancy of nearly 10°, with one showing a difference of 26°. In four cases, varus elbows with negative angles were observed (Table 4).

**Table 4:** Carrying angles and Flynn’s criteria

Patients		Carrying angle		Rating (Flynn's criteria)
		Uninjured side	Fractured side (N.V. 12–16°)	
1	B.A	12°	−3°	Poor
2	B.M	12°	14°	Excellent
3	B.L	0°	1°	Excellent
4	C.L	14°	20°	Good
5	C.E	25°	20°	Good
6	C.G	10°	0°	Fair
7	D.F	20°	15°	Good
8	E.H	15°	1°	Fair
9	E.V	20°	5°	Poor
10	F.S	20°	10°	Fair
11	F.D	22°	20°	Excellent
12	F.SG	15°	10°	Good
13	F.A	15°	7°	Fair
14	M.A	10°	−5°	Poor
15	M.D	19°	14°	Good
16	M.G	5°	7°	Excellent
17	P.P	14°	5°	Good
18	P.D	16°	10°	Good
19	R.So	5°	−10°	Poor
20	R.Sa	14°	−12°	Poor
21	R.St	10°	13°	Excellent
22	S.G	19°	22°	Excellent
23	T.M	10°	10°	Excellent
24	T.C	15°	15°	Excellent
25	V.F	10°	10°	Excellent
26	Z.N	17°	7°	Fair
27	T.R	15°	12°	Excellent
Average	14°	8°		
SD	5.57	8.89		
*p* value	***0.00034***			

Bolditalic value indicates statistically significant *p* value

*N.V.* Normal value

Regarding the values observed for extension, pronation, and supination, although there are variations between the uninjured and fractured sides (differences in the mean values), they are not significant enough to warrant a statistical test (Tables 5, 6, 7, and 8).

**Table 5:** Comparision of the functional flexion between the uninjured side and fractured side treated respectively conservatively and with K-wires

ROM—flexion	Uninjured side	Fractured side with cast
Average	142°	130°
SD	4	11.6°
*p* value	***0.016***	

	Uninjured side	Fractured side with K-wires
Average	142°	140°
SD	4.55	7.07
*p* value	0.1225	

Bolditalic value indicates statistically significant *p* value

**Table 6:** Carrying angle measurements for both groups treated with a cast and K-wires: surgically treated patients exhibited significantly superior outcomes in maintaining an acceptable carrying angle compared to the other group

Carrying angle	Uninjured	Injured side with cast
Average	13°	3°
SD	5.22°	10°
*p* value	***0.022***	

	Uninjured	Injured side with K-wires
Average	15°	10°
SD	5.78°	7.77°
*p* value	***0.0068***	

Bolditalic value indicates statistically significant *p* value

**Table 7:** The hand grip dynamometer tests showed no significant decreases in strength in patients treated conservatively. Still, a noticeable reduction in strength between the two limbs was found in the surgical group

Grip strength	Uninjured	Fractured side with cast	Grip strength	Uninjured	Fractured side with K-wires
Average	15.84	14.63	Average	17.01	15.84
SD	8.27	6.68	SD	8.00	7.62
*p* value	0.17		*p* value	0.07

**Table 8:** A final comparison of clinical outcomes correlating the fracture type with the treatment administered in Gartland type II fractures

G II type with K-wire pinning			G II type treated with cast		
ROM—flexion	Uninjured side	Fractured side	ROM—flexion	Injured side	Fractured
Average	142°	141°	Average	142°	130°
SD	4.13°	6.18°	SD	4.27°	11.65°
*p* value	0.85		*p* value	***0.01***	

Carryng angle			Carryng angle		
Average	15°	11°	Average	13°	3°
SD	6.36°	7.99°	SD	5.22°	10.02°
*p* value	0.10		*p* value	***0.02***	

Bolditalic value indicates statistically significant *p* value

The clinical outcomes obtained from the scores were then compared with the type of treatment used, differentiating between closed reduction and casting and closed reduction with percutaneous cross-pinning (considering both two pins and three pins). There were eight patients undergoing the former treatment and 19 patients undergoing the latter one. Regarding the functional flexion angle, according to Flynn's criteria, 50% of the patients with reduction and casting show poor results, while the other 50% show acceptable results, with 37% showing excellent and 13% showing good results. The mean for the healthy side is 142° ± 4°, compared to 130° ± 11.6° for the fractured limb. Statistical analysis reports a* p* value below 0.05, indicating a significant correlation between the reduction in the flexion angle and this type of treatment. In comparison, 21% of the patients treated with reduction and fixation with crossed K-wires exhibit modest results and 79% exhibit satisfactory results. The mean for the healthy side is 142° ± 4.5°, compared to 140° ± 7° for the fractured limb. Finally, statistical analysis reports a value of 0.1, which is considered to be not significant, indicating no correlation between surgical treatment and flexion angle.

In patients treated with reduction and casting, Flynn's carrying angle criteria showed that 38% of the patients presented poor, 12% presented fair, and 50% presented good to excellent results. Statistical analysis revealed a significant correlation between a decreased carrying angle and conservative treatment (*p* < 0.02). Conversely, 11% of the patients treated with crossed pinning had poor, 21% had fair, and 68% had good to excellent outcomes, with a significant *p* value of 0.0068.

These findings suggest that surgically treated patients exhibited significantly superior outcomes in maintaining an acceptable carrying angle compared to the other group.

In reference to the hand grip dynamometer tests, no significant decreases in strength were observed in patients treated conservatively. The mean grip strength was 16 ± 8.3 kg for the healthy side and 15 ± 6.7 kg for the injured side, with a calculated *p* value of 0.17. Conversely, in patients undergoing reduction with internal fixation, a noticeable reduction in strength between the two limbs was found, with a mean of 17.01 ± 8 kg for the healthy side and 15.84 ± 7.6 kg for the fractured side. The statistical analysis using Student's *t*-test yielded a *p* value of 0.07.

A final comparison of clinical outcomes was conducted by correlating the fracture type with the treatment administered. Only Gartland type II fractures were considered, as the Gartland type III fractures were all treated with reduction and fixation. Within this subset, clinical outcomes were assessed based on the type of treatment.

The results show that the 11 children treated with closed reduction and percutaneous pinning for G II fractures (10 G IIb and one G IIa) exhibited a mean flexion angle of 141.6° ± 4.1° for the healthy limb and 141.2° ± 6.2° for the fractured limb during follow-up. Flynn's evaluation criteria show that 91% of the cases achieved good/excellent results, with the remaining 9% achieving satisfactory results. Analysis of the* t*-test for strength did not show significance (*p* = 0.85). Conversely, the 8 patients treated conservatively for G IIa fractures presented a mean flexion angle of 141° ± 4.3° for the healthy limb and 130° ± 11.6° for the fractured limb. Flynn's criteria show that 50% of the cases had unsatisfactory results. Statistical analysis confirms that the surgically treated patients had better medium-term outcomes, with statistically significant differences in both functional and cubital angles (*p* < 0.05), while the tests for strength, extension, and pronation–supination did not yield significant results.

## Discussion

In the studied series, the overall functional outcome was favorable after an average follow-up of 3.3 years, as assessed by the m-DASH score. Specifically, 64.5% of children achieved an excellent outcome, while 35.4% had a positive score with low values, indicating only slight disabilities or symptoms. It is important to note the challenge of comparing pediatric patients, as parental influence can significantly impact the behavior and self-confidence of children. Regarding elbow range of motion (ROM) in the medium term, the results were satisfactory according to Flynn’s criteria: 85% of patients demonstrated good outcomes (with ROM reductions of < 15°). A higher incidence of unsatisfactory outcomes was observed in displaced G II fractures, where 22% of patients had unfavorable outcomes compared to children with G III fractures, who achieved total success. The rationale for these findings can be attributed to the divergent treatment approaches employed. Specifically, all G III fractures underwent surgical intervention, whereas within the G II fracture cohort, certain patients, specifically those with G IIa fractures, underwent conservative treatment with closed reduction and casting. The statistical analysis comparing the clinical outcomes of type IIa fractures treated conservatively versus type IIb fractures treated surgically with percutaneous pinning reveals a significant difference: patients treated conservatively exhibited inferior mid-term functional outcomes in terms of both flexion (*p* < 0.01) and carrying angle (*p* < 0.02) compared to those managed surgically.

A comprehensive analysis of the literature confirms a consensus regarding the surgical management of Gartland type III SCHF [15, 16]. Percutaneous pinning is considered the optimal treatment for displaced SCHF due to its safety and reliability, with a reduced risk of cubitus varus deformity [17]. Sinikumpu et al. demonstrated excellent long-term clinical outcomes at 10 years following surgical treatment of G I fractures [18].

Although the treatment approach for type III fractures is widely acknowledged in the scientific literature, ongoing debate exists regarding the optimal management of Gartland type II. In a comprehensive consecutive series comprising 69 children, Skaggs et al. demonstrated the effectiveness of stabilizing Gartland type II fractures through lateral pinning. Significantly, no iatrogenic ulnar nerve injuries or loss of reduction was observed [19]. In a case–control study comparing the outcomes of type IIb and type III fractures, 30 cases treated with closed reduction and casting were contrasted with 30 cases treated with closed reduction and fixation using crossed K-wires. The authors concluded that surgically managed patients showed superior functional and cosmetic outcomes as well as better preservation of range of movement [20]. An epidemiological study from 2017 revealed an increasing trend in the incidence of type II supracondylar humerus fractures as well as their surgical treatment with lateral pinning [21]. In 2019, Kropelnicki et al. published a review on the good management of pediatric SCHF in accordance with the British Orthopaedic Association Standards for Trauma. The authors recommend surgical treatment involving closed reduction and percutaneous pinning with two K-wires (there is no consensus about the best configuration of the K-wires) as the standard approach for nearly all G IIb type fractures and all type G III fractures [22]. On the other hand, many authors support the efficacy and effectiveness of conservative management for Gartland type II fractures; for instance, in 2004, Parikh et al. proposed closed reduction and casting without pinning as an initial viable approach for managing displaced type II fractures in patients who are able to undergo regular weekly follow-up radiographs. This recommendation was supported by favorable outcomes, with 72% of the reductions deemed satisfactory among a cohort of 25 patients [23]. In a recent study published in 2021, the authors highlighted favorable outcomes of reduction and casting for Gartland type II supracondylar humerus fractures: among the 77 type II fractures treated initially with reduction and casting, 77% maintained their anatomical alignment. Any loss of alignment typically occurs within the first week post-treatment [24]. In our study, a similar event occurred: a patient with a G IIa fracture initially underwent reduction and casting; however, at the radiographic follow-up after 1 week, there was evidence of displacement, leading to a conversion to surgical pinning treatment.

Sisman et al. argue that outcomes for non-surgically treated type IIb fractures are comparable to those of conservatively managed type IIa fractures. Their study reveals satisfactory treatment outcomes for all type II SCHF when the reduction remains stable within the first week following closed reduction and casting. Critically, no instances of reduction loss were observed beyond the initial week [10]. In a more recent study, Danjiang et al. compared and assessed the effectiveness of conservative versus surgical interventions for G II SCHF. They treated 142 patients, dividing them into two groups: group A received initial conservative treatment, while group B underwent initial surgical management. The results showed no significant differences between the two groups' fracture healing times and Flynn scores. However, the conservative treatment demonstrated superior functional recovery times compared to surgery [25]. The limitations of our study include the small number of patients and the absence of long-term follow-up. Despite the limited sample size, no long-term complications related to the positioning of the K-wires, infections, or ulnar nerve injuries were observed in our cohort. The incidence of pin infections is noted to be extremely low in the literature [26]. To reduce the incidence of iatrogenic ulnar nerve injuries during percutaneous fixation, surgeons propose possible solutions such as performing a mini-open approach, using electrical stimulation of the nerve during surgery, positioning the patient prone on the operating table, or inserting two lateral wires without the medial K-wire [27–29].

The literature presents studies discussing the advantages and disadvantages of positioning patients prone or supine in the operating room. A notable complication associated with the supine position is ulnar nerve injury, which typically occurs during medial K-wire insertion when the ulnar nerve undergoes anterior displacement during the elbow hyperflexion reduction maneuver. However, there is insufficient evidence to establish the superiority of either prone or supine positioning in terms of clinical and functional outcomes. The choice between these positions largely hinges on the surgeon's preferences and the anesthesiologist's experience [30]. In our clinical practice, we prefer to place the patient in the prone position, occasionally opting for a mini-open incision to avoid any ulnar nerve injury if there is significant elbow swelling. In light of the good clinical outcomes elucidated in this study in the surgical treatment of Gartland type II and III SCHF, and considering the arguments advanced in contemporary literature, the authors maintain a preference for a surgical approach with closed reduction and pinning. The authors acknowledge the limitation of the relatively small sample size in this study. To address this limitation, it will be essential to continue expanding the case studies and to persist in recruiting future patients. A prospective enrollment allowing for a comparative analysis between two patient groups stratified by treatment type would be even more effective and significant. In addition, it would be interesting to combine clinical evaluation with imaging analysis for a more comprehensive understanding and definition of the management and outcomes of SCHF treatment. This approach should incorporate measurements from pre- and post-treatment radiographs to assess the radiological progression of healing and functional recovery from a clinical perspective.

## Data Availability

The datasets used and analyzed during the current study are available from the corresponding author on reasonable request. In order to ensure confidentiality of data and to avoid data loss or manipulation, precautionary measures have been taken: data availability is restricted to authorized members only. These authorized members are Prof. Magnan MD, Prof. Samaila MD, L. Auregli MD, and L. Pezzè MD. All information about the patients enrolled in the study is stored on a secure server, and database access is protected through a password that periodically changes. Only authorized members have the password. Paper-based material about clinical evaluations is kept in a cabinet in the office of Professor Samaila.

## Abbreviations

SCHF: Supracondylar humeral fractures
G I: Gartland I
G II: Gartland II
G III: Gartland III
AUC: Appropriate Use Criteria
m-DASH: Modified Disabilities of the Arm, Shoulder, and Hand
